# Expression of Human Endogenous Retroviruses in Peripheral Blood of Acute and Chronically HIV-Infected Subjects and Effect of Antiretroviral Therapy

**DOI:** 10.3390/ijms27136025

**Published:** 2026-07-04

**Authors:** Elisabetta Lazzari, Gabriella Rozera, Lucrezia Pierfederici, Daniele Pietrucci, Daniele Maria Papetti, Lavinia Fabeni, Flavia Smoquina, Giulia Berno, Federica Forbici, Valentina Mazzotta, Roberta Gagliardini, Andrea Antinori, Giovanni Chillemi, Fabrizio Maggi, Isabella Abbate

**Affiliations:** 1 Laboratory of Virology, National Institute for Infectious Diseases Lazzaro Spallanzani IRCCS, 00149 Rome, Italy; elisabetta.lazzari@inmi.it (E.L.); lavinia.fabeni@inmi.it (L.F.); flavia.smoquina@inmi.it (F.S.); giulia.berno@inmi.it (G.B.); federica.forbici@inmi.it (F.F.); fabrizio.maggi@inmi.it (F.M.); isabella.abbate@inmi.it (I.A.); 2 Bioinformatics Research Unit in Infectious Diseases, National Institute for Infectious Diseases Lazzaro Spallanzani IRCCS, 00149 Rome, Italy; lucrezia.pierfederici@alumni.uniroma2.eu (L.P.); giovanni.chillemi@inmi.it (G.C.); 3 Department of Experimental Medicine, University of Rome “Tor Vergata”, 00133 Rome, Italy; 4 Department for Innovation in Biological, Agro-Food and Forest Systems—DIBAF, University of Tuscia, 01100 Viterbo, Italy; daniele.pietrucci@unitus.it; 5 Department of Informatics, Systems and Communication, University of Milano-Bicocca, 20126 Milan, Italy; daniele.papetti@unimib.it; 6 Bicocca Bioinformatics Biostatistics and Bioimaging B4 Center, Department of Medicine and Surgery, University of Milano-Bicocca, 20900 Monza, Italy; 7 Clinical Department, National Institute for Infectious Diseases Lazzaro Spallanzani IRCCS, 00149 Rome, Italy; valentina.mazzotta@inmi.it (V.M.); roberta.gagliardini@inmi.it (R.G.); andrea.antinori@inmi.it (A.A.)

**Keywords:** HIV, human endogenous retroviruses, transcriptomics, antiretroviral treatment

## Abstract

Human endogenous retroviruses (HERVs) originate from ancient retroviral integration into the primate germline. Although most are defective proviruses, the most recently endogenized groups, like the HERV-K family, retain intact ORFs encoding retroviral proteins. HERVs usually remain transcriptionally silent, yet this status is reversible. Multiple HIV-HERV interactions, mainly mediated by the HIV Tat protein, lead to HERV transcription and protein production. The present study investigates HERV-K transcription in particular of Human MMTV-like (HML) group-2 and 6 in peripheral blood of people with HIV (PWH). Using different experimental approaches—such as single-cell and plasma transcriptomics-, we found that HERV-K transcripts may be detected during both acute and chronic phases of the infection, with HML-6 showing higher expression compared to HML-2, predominantly within myeloid cells. Effective combined antiretroviral therapy (cART) was able to significantly reduce HML-6 transcription, regardless of whether the treatment was initiated in the acute or late chronic phases of HIV infection. Notably, chronic infections showed higher HML-6 transcript levels compared to acute infections in both naïve and successfully cART-treated subjects, potentially associated with persistent immune dysregulation observed in chronic HIV infection, although a direct causal role of HML-6 expression remains to be established.

## 1. Introduction

Human endogenous retroviruses (HERVs) originate from ancient retroviral integration within the primate germline. Successive waves of integration by different retroviruses, and subsequent amplification over the course of evolution, led to the accumulation of a huge number of proviral copies in the human genome, which currently represent around 8% of the human cellular DNA. The majority of these sequences are defective proviruses with mutations or deletions, arising primarily from recombination events among the LTRs [1]. However, the most recently endogenized groups, including the Human Mouse Mammary Tumor (MMTV)-like group (HML), from HML-1 to HML-10, in the HERV-K family, frequently retain intact ORFs encoding all canonical retroviral proteins. In addition to genetic disintegration from recombination events, most HERVs are kept transcriptionally silent via a dedicated repressive DNA methylating machinery, fostering a heterochromatic epigenetic state. This epigenetic control, established in the pre-implantation embryo and subsequently maintained across development and adult life in most tissues, can be reversible. Accordingly, both HERV transcripts and proteins have been found in autoimmune, neoplastic, and neurodegenerative disorders, possibly contributing to immune dysregulation and pathogenetic mechanisms of the diseases [2,3]. However, recent increasing evidence implies that HERV expression may be present among normal tissues, following body site-specific patterns as well as biological factors such as sex, ethnicity, and age [4].

Infectious diseases may induce HERV transcription, and HIV infection has been the most widely studied in this regard [5,6]. Multiple interactions between HIV and HERVs have been described, most of which are mediated by HIV Tat protein and involve cellular transcription factors such as NF-κB/NF-AT, Toll-like receptor 4 (TLR4) pathway, and alterations in chromatin structure [7]. In addition, HERV-K gag is capable of co-assembling with HIV-1 gag, interfering with both HIV-1 gag assembly and the subsequent release of viral particles [8]. The HIV rev protein can functionally interact with many HERV-K Rec Responsive Elements present in the human genome, leading to the export of some of the HERV-K proviral mRNAs. It also has the potential to change the expression of non-viral genes [9]. HIV-1 vif and vpu appear to promote HERV-K transcription and viral particle release, respectively—vif counteracting APOBEC [10] and vpu neutralizing tetherin [11]. However, the effect of HIV proteins on HERV expression can vary considerably, not only between different HERV families and groups [12], but also between different proviruses within the same group [13], depending on the biological context and the cell types. On the other hand, recent studies have shown that infection with different HIV-1 subtypes exhibits different effects on HERV-K transcription [14], again in a cell type-dependent fashion [12]. In the context of HERV-K detection in clinical samples, Contreras-Galindo, R. et al. [15] were the first to report HERV-K viral-like particle detection in the blood of an HIV-infected individual.

More recently, Mantovani et al. [12] showed that HIV is able to alter the expression of various HERV families in the peripheral blood of HIV-infected individuals. Elevated HERV-K HML-2 *gag* and *env* mRNA levels were reported during late stages of HIV infection by Ormsby et al. [16]. Furthermore, HERV-K titers were usually undetectable in patients with successful highly active antiretroviral treatment (HAART) or, when present, they remained below 5000 copies/mL. Additionally, HERV-K RNA was detected consistently in patients who failed to respond to HAART, both before and after HIV-1 rebounds [17]. HIV elite controllers showed a significantly lower expression of HERV-K in peripheral blood mononuclear cells (PBMC), compared to both healthy controls and cART-treated people with HIV (PWH) [6]. Additionally, it has been reported that HERV LTRs contribute to approximately 15–30% of enhancer elements in immune cells, especially near genes involved in immune response and cellular stress [18,19], potentially driving the abnormal immune activation in HIV-infected subjects. Taken together, these studies suggest that HERV expression may vary under different HIV disease stages, immune activation status, and cART.

The present study investigates HERV-K transcription in peripheral blood, both at the single-cell level and in plasma, of individuals with acute and chronic HIV infection. It also evaluates whether anti-HIV effective cART is equally effective in reducing HERV-K levels regardless of whether treatment is started during the acute or late chronic phase of the infection.

## 2. Results

### 2.1. Clinical and Virological Characterization of Studied Subjects

HERV-K transcription was studied at the single-cell level on peripheral blood mononuclear cells (PBMC) collected from two PWH representative of different stages and conditions of HIV infection. S1 was a 37-year-old male with a primary HIV infection, as established by a serological avidity test [20], while S2 was a 53-year-old male with a chronic HIV infection, on cART (Darunavir/cobicistat plus tenofovir alafenamide) started again two months before sample collection, after a period of non-adherence. At the moment of the analysis, S1 and S2 subjects displayed a plasma viremia of 4.84 and 4.59 Log HIV-1 RNA copies/mL, respectively. CD4 T-cell counts were 323 and 238 cells/mm^3^, while CD4/CD8 ratios were 0.63 and 0.16, respectively.

To evaluate the effect of cART in reducing HERV-K levels, regardless of the stage of HIV infection when antiviral therapy was started, other participants were selected from two groups: acute HIV-infected (AHI) subjects and chronically HIV infected (CHI) subjects, presenting with advanced HIV disease (late presenters). All subjects had samples at the moment of serodiagnosis (T0) and after one year of effective cART (T1), started soon after serodiagnosis. At T1, all individuals reached complete viral suppression, defined as showing an HIV-1 RNA <50 copies/mL in plasma samples. Plasma samples from five healthy donors (HD) were used as controls.

All AHI and HD participants were male, with a median (IQR) age of 51 (35–57) and 38 (31–50) years, respectively. The CHI group consisted of five males and two females, with a median (IQR) age of 55 (48–75) years. HD tested negative for HIV-1, and all subjects tested negative for hepatitis markers, except for two CHIs, which were positive for HBV core antibodies but negative for HBV-DNA. At baseline, AHI and CHI displayed median (IQR) Log HIV-1 RNA of 6.08 (4.68–7.74) and 5.66 (4.28–6.45) cp/mL, respectively; by T1, all PWH were virologically suppressed. At T0, AHI and CHI subjects displayed CD4 median (IQR) values of 349 (204–669) and 122 (38–190) cells/mm^3^, while at T1 the values rose to 920 (445–964) and 399 (234–514) cells/mm^3^, respectively. At baseline, median (IQR) CD4/CD8 ratios were 0.7 (0.15–1.09) and 0.15 (0.10–0.28) in AHI and CHI. At T1, ratios were 1.10 (0.43–1.53) and 0.46 (0.41–0.55) in AHI and CHI, respectively.

### 2.2. Identification of Peripheral Blood Cell Types Harboring HERV-K Transcripts

After quality control and filtering processes, a total of 8063 single-cell transcriptomes were obtained from S1 and S2 PBMC, corresponding to a median (IQR) of genes detected per cell of 1893 (1424–2874).

**Figure 1:**
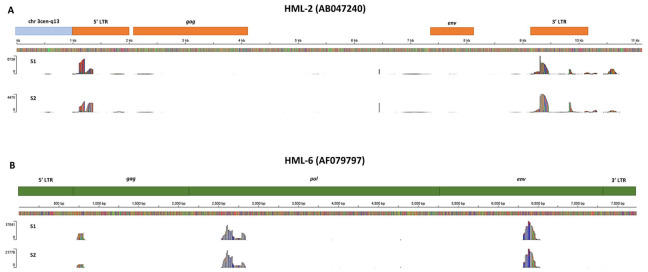
**Visualization of aligned HERV-K viral transcripts in PWH within acute (S1) and chronic (S2) infection**. (**A**), Alignment of viral transcripts found in PBMCs of patients S1 and S2 against the HERV-K HML-2 genome (Gene Bank: AB047240) performed on the IGV web app (last accessed on 26 May 2026). The reference genome is represented as a colored line. Viral genes are represented as orange boxes, with each name written above, according to the genomic positions annotated on the reference genome. Human genomic sequences are displayed as blue boxes, with each name written above, according to the genomic positions annotated on the reference genome. The coverage of the alignment is represented below the reference genome, and its height is displayed on the left. (**B**), Alignment of viral transcripts found in PBMCs of patients S1 and S2 against the HERV-K HML-6 genome (Gene Bank: AF079797), performed on the IGV web app (last accessed on 26 May 2026). The reference genome is represented as a colored line. Viral genes are represented as green boxes, with each name written above, according to the genomic positions annotated on the reference genome. The coverage of the alignment is represented below the reference genome, and its height is displayed on the left.

Considering HERV-K transcripts, a number of LTR, *gag*, and *env* HML-2 and HML-6 transcripts were found in PBMC of both subjects. In Figure 1, the coverage of viral transcripts found in S1 (Figure 1A) and S2 (Figure 1B), aligned to reference genomes of HML-2 and HML-6, is reported. In Figure 1A, it can be noted that, in both S1 and S2 samples, the highest coverage was found in the LTR regions of HML-2, while in the *gag* and *env* regions the coverage was lower. On the other hand, Figure 1B highlights a different picture: the highest coverage, in the two samples, was reported in both *pol* and *env* regions, while the *gag* region was characterized by lower coverage. Alignment metrics are described in Supplementary Table S1.

**Figure 2:**
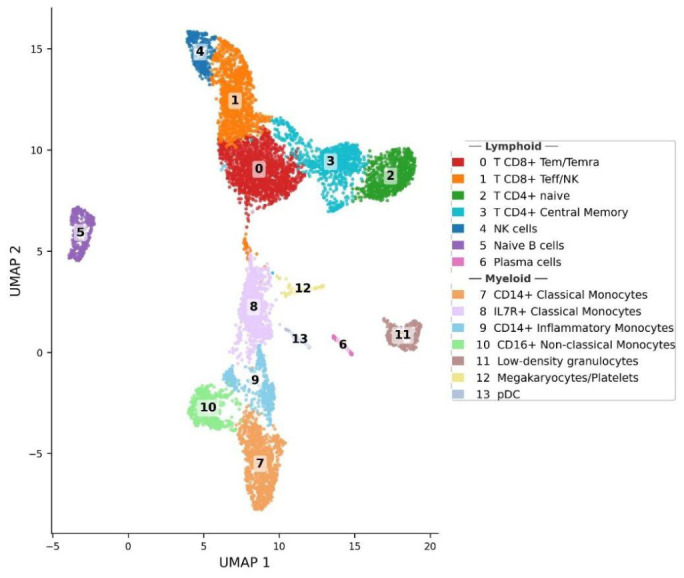
**Cell type annotation of PBMC**. UMAP visualization of single-cell RNA sequencing data from PBMCs of S1 and S2 subjects. Fourteen distinct cell clusters were identified, spanning both lymphoid and myeloid lineages. Each cluster, shown with different colors, corresponded to a specific cell type listed in the insert on the right.

In order to identify which peripheral blood cell type harbored the retroviral transcripts, unsupervised clustering analysis and cell type annotation were performed as described in the methods. These analyses highlighted 14 different clusters, each corresponding to a specific cell type represented in the UMAP plot (Figure 2). Table 1 presents the numerosity of the different cell types identified in S1 and S2 subjects.

**Figure 3:**
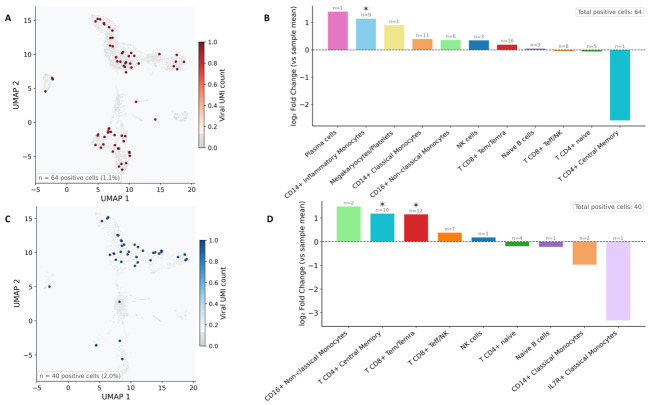
**HML-2 transcript distribution and log_2_ fold change enrichment across PBMC of S1 and S2.** (**A**) UMAP showing the distribution of HML-2 viral transcripts in S1. Positive cells are colored from gray to red according to the viral UMI count. The number of positive cells is indicated below in the graph. (**B**) Bar plot showing the log_2_ fold change enrichment of HML-2 transcripts by cell type in S1. Statistically significant enrichments are highlighted with a star. Colors represent the different cell populations identified. (**C**) UMAP showing the distribution of HML-2 viral transcripts in S2. Positive cells are colored from gray to dark blue according to the viral UMI count. The number of positive cells is indicated below in the graph. (**D**) Bar plot showing the log_2_ fold change enrichment of HML-2 transcripts by cell type in S2. Statistically significant enrichments are highlighted with a star. Colors represent the different cell populations identified.

**Figure 4:**
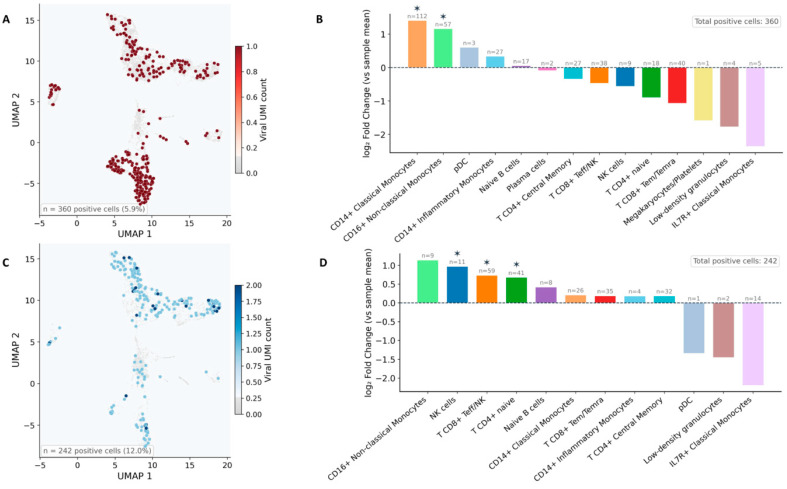
**HML-6 transcript distribution and log_2_ fold change enrichment across PBMC of S1 and S2.** (**A**) UMAP showing the distribution of HML-6 viral transcripts in S1. Positive cells are colored from gray to red according to the viral UMI count. The number of positive cells is indicated below in the graph. (**B**) Bar plot showing the log_2_ fold change enrichment of HML-6 transcripts by cell type in S1. Statistically significant enrichments are highlighted with a star. Colors represent the different cell populations identified. (**C**) UMAP showing the distribution of HML-6 viral transcripts in S2. Positive cells are colored from gray to dark blue according to the viral UMI count. The number of positive cells is indicated below in the graph. (**D**) Bar plot showing the log_2_ fold change enrichment of HML-6 transcripts by cell type in S2. Statistically significant enrichments are highlighted with a star. Colors represent the different cell populations identified.

Figure 3A,C and Figure 4A,C show the distribution of HML-2 (Figure 3) and HML-6 (Figure 4) transcripts, respectively, across PBMCs of S1 and S2. Figure 3B,D and Figure 4B,D present bar plots showing the log_2_ fold change enrichment of viral transcripts by cell type, with statistically significant enrichments identified using a one-sided Mann–Whitney U test with Benjamini–Hochberg FDR correction (adjusted *p* < 0.05). Consistent with the higher amounts of HML-6 transcripts mapped to the reference sequence compared to HML-2, these plots reveal a higher proportion of cells harboring HML-6 transcripts relative to HML-2 in the PBMC of both S1 and S2 (5.9 and 12.0 vs. 1.1 and 2.0%), respectively. Regarding the cell types harboring HERV-K transcripts, subject S2 displays statistically significant enrichment in lymphoid-lineage cells (T CD4, T CD8 and NK lymphocytes), which are not observed in S1, where HERV-K transcripts show statistically significant enrichment only in cells of myeloid origin.

### 2.3. Expression Levels of HML-6 env Transcripts in Plasma Samples and Effect of Antiretroviral Treatment

As described above, single-cell analysis highlighted that the most represented HERV-K transcripts in PBMC of either acute- or chronically infected subjects aligned to the HML-6 *env* region. For this reason, to evaluate both possible differences in retroviral endogenous transcription among different HIV infection stages and the effect of cART, we focused on HML-6 *env* transcript levels.

At serodiagnosis (T0), HIV-1 viremia did not differ between AHI and CHI: 6.08 (4.68–7.74) and 5.66 (4.28–6.45) Log copies/mL. Moreover, the correlation between HML-6 transcript expression and HIV-1 RNA did not reach statistical significance (r = 0.376, *p* = 0.186).

**Figure 5:**
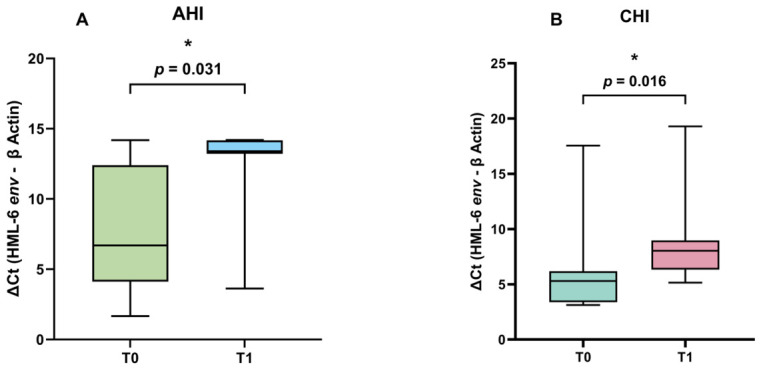
**HML-6 *env* expression in AHI and CHI during cART.** (**A**) Box plots display the distribution of ΔCt (HML-6 *env*—β Actin) in AHI at T0, in green, and at T1, in light blue. (**B**) Box plots display the distribution of ΔCt (HML-6 *env*—β Actin) in CHI at T0, in teal, and at T1, in pink. In both panels, on the *y* axis, HML-6 *env* expression values are displayed as values of ΔCt (HML6 *env—*β Actin); the median is highlighted as a bold black line inside each plot. *p*-value referring to the difference between T0 and T1 is displayed above; significative *p*-values (*p* < 0.05) are indicated with a star.

HML-6 transcripts were undetectable in HD. On the other hand, in both AHI and CHI groups, expression levels significantly decreased (increase in ΔCt values) after one year of effective cART (T1), with median (IRQ) ΔCt values of 6.69 (4.11–12.40) vs. 13.40 (13.21–14.17) in AHI, and 5.29 (3.38–6.18) vs. 8.03 (6.33–8.97) in CHI, at T0 and T1, respectively (*p* = 0.016 and *p* = 0.031) (Figure 5A,B). This decline corresponded to a fold change (FC) reduction of 24 times in the AHI group and 6 times in CHI, respectively. Although not reaching statistical significance, HML-6 transcript levels at both T0 and T1 were higher in CHI than in AHI.

## 3. Discussion

In the present study, using different experimental approaches—such as single-cell and plasma transcriptomics- we found that HERV-K transcripts may be detected in PWH during both acute and chronic infection, with HML-6 showing higher expression levels compared to HML-2. No correlation was found between HML-6 transcript levels and HIV-1 RNA in cART-naïve subjects. However, effective cART may significantly reduce HML-6 transcription, regardless of whether treatment is initiated in the acute or late chronic phases of HIV infection. Nevertheless, a trend toward higher amounts of HML-6 transcripts was observed in chronic compared to acute infection, in both naïve and successfully cART-treated subjects.

HERV gene expression is strictly regulated by the human organism through epigenetic mechanisms evolved during coexistence with the human host. HERVs are systematically transcribed in a stage-specific manner during early human embryogenesis, and their expression acts as an indicator of cellular identity. It was believed that HERV expression was mainly related to pathological conditions, such as neoplastic, autoimmune and infectious diseases [21,22,23], but increasing evidence is showing that there are plenty of expressed HERVs in normal tissues, following body site-specific patterns, as well as biological factors such as sex, ethnicity, and age [4].

Among the infectious diseases associated with HERV reactivation, HIV infection has been the most studied; however, the peripheral blood cell type primarily involved in this reactivation remains to be defined. Previous studies examining CD4 T, CD8 T, B lymphocytes and monocytes from PWH, sorted by flow cytometry, failed to identify statistically significant HERV-K (HML-2) transcript up-regulation in any of these cell populations compared to uninfected controls; nevertheless, monocytes displayed the greatest difference in HERV expression between PWH and controls Bhardwaj et al. [24].

In our limited experience regarding only one acute and one chronic HIV-infected subject, in accordance with Bhardwaj and Mantovani et al. [12,24], we found that cells of monocytic origin seem to be the main cell type in which HIV presence may have an effect on HERV expression. In addition, and in only the PBMC of the chronically infected subject, HERV-K transcripts were also found in CD4 naïve and central memory, CD8 effector memory RA positive (TEMRA) and NK cells. Moreover, as reported by Grandi et al. [13], in the PBMC of these two subjects, HML-6 expression has been found to be higher than that of HML-2 in PWH.

It has to be underlined that, in the present study, cells harboring HERV-K transcripts outnumbered those displaying HIV transcripts by two to three orders of magnitude; the latter were restricted to CD4 T cells, consistent with our previous findings on subject S1 [25]. This observation suggests that HIV-induced up-regulation of HERV is not confined to HIV-infected cells, but is more probably exerted by soluble factors acting on bystander cells.

While no drugs are designed to specifically target HERVs, a number of studies have been performed in order to determine if HIV-specific antiretroviral drugs could affect HERV infection and viral production. Tyagi et al., using a pseudotyped HERV-K with VSV-G to infect HeLa cells, found that RT inhibitors Abacavir and Zidovudine, and integrase inhibitor Raltegravir could effectively block HERV-K infection and production. However, protease inhibitors were not as effective as RT and integrase inhibitors [26]. Mantovani et al. [12], analyzing publicly available human transcriptomic datasets, reported an increased expression of multiple HERV families in the PBMCs of HIV-infected individuals undergoing combination antiretroviral therapy, compared to healthy donors. These findings highlighted considerable differences among different HERV family members, and suggested that antiretroviral therapy may not completely suppress HERV gene expression.

In PWH who initiate cART at the late chronic stage, with critically low CD4 T cell counts, it is difficult to obtain an immunological recovery with a consistent reduction in immune activation, even when a virological response is achieved. This phenomenon, referred to as “the immune dysregulation legacy effect”, is based on the observation that pre-ART strongly correlates with on-ART inflammatory levels, suggesting that pathogenic mechanisms that occurred prior to therapy initiation are the predominant drivers of long-lasting immune dysregulation [27].

The findings of the present study, conducted on PWH who achieved virological suppression after one year of cART, suggest that antiretroviral therapy is effective in reducing HML-6 expression, even when initiated in late HIV-diagnosed, chronically infected subjects. However, other host-specific mechanisms, either acting independently or in synergy with ART, cannot be ruled out as factors contributing to the decline of HML-6 levels during the natural course of the disease.

In the present paper, it is shown that HML-6 transcript levels, although not reaching statistical significance, remained higher compared to cART-treated AHI individuals. A larger sample size of samples comprising PWH diagnosed and treated at very different stages of HIV infection might confirm the observed trend and provide further insights into the different HML-6 expression dynamics across the natural history of HIV infection, together with the role of cART. Isolating the direct effect of individual viruses on inflammatory markers was not possible in our study, as the experimental design featured HIV infection as a simultaneous driver of immune activation alongside HERV transcripts. Consequently, future in vitro studies investigating Tat and cytokine-mediated HERV-K induction are warranted to distinguish between simple association and true mechanistic causality.

## 4. Materials and Methods

### 4.1. Study Population

Plasma samples from the 7 AHI subjects were taken from the biobank repository of the INMI observational cohort of primary infection SIREA (Studio di Coorte osservazionale di pazienti con sindrome retrovirale acuta). The study was approved by the National Institute for Infectious Diseases (INMI) Lazzaro Spallanzani Ethical Committee on 18 February 2014. Diagnostic residual samples were used for S1, S2 and late-presenting PWH (CHI).

Serodiagnosis of AHI patients was based on either the combination of an HIV Ab/Ag Combo positive and an immunoblot negative assay (Fiebig II/III stage) or an HIV Ab/Ag Combo positive and immunoblot indeterminate assay (Fiebig IV stage), according to the WHO criteria for the Western blot confirmatory assay, i.e., two env reactive bands. CHI patients were consecutively enrolled as they presented for clinical evaluation. Inclusion/exclusion criteria were as follows: ART-naïve HIV-infected, aged 18 years or older, newly diagnosed with HIV-1 infection, presenting with a confirmed AIDS-defining event, and/or CD4 cell count below 200 cells/µL. Antiviral therapy was initiated in both groups of patients within a week, at the latest, from serodiagnosis and consisted of AHI in tenofovir/emtricitabine and either bictegravir or dolutegravir, whereas CHI was based only on bictegravir/emtricitabine/tenofovir. Plasma HIV-1 RNA was measured by Aptima™ HIV-1 Quant Dx Assay (Hologic, Inc., San Diego, CA, USA). All subjects were requested to sign an informed consent form.

### 4.2. Single Cell Transcriptomics

To identify HML-2 and HML-6 transcripts, single-cell transcriptomics was carried out on PBMCs collected from peripheral blood of 1 acute naïve to antiretrovirals HIV-infection (S1) and 1 chronic on ART HIV-infection (S2), and subsequently washed. Viable cells were measured using trypan blue. Approximately 5000 cells per sample were processed for reverse transcription and barcoding using 10X Chromium Single Cell reagents and platform (10X Genomics, Pleasanton, CA, USA). Following the Single Cell 3′ Reagent Kits v3 protocol (10X Genomics), cDNA libraries were generated from every single barcoded cell, and each library was fragmented and ligated to adapters for sequencing on the Illumina platform NovaSeqTM 6000 System (Illumina, San Diego, CA, USA). At least 20,000 read pairs per single barcoded cell were used for the analysis.

### 4.3. Bioinformatics Pipeline

#### 4.3.1. Pre-Processing Analysis

The presence of HML-2 and HML-6 transcripts was investigated using a specifically established bioinformatic pipeline. Briefly, Cell Ranger software (version 7.1.0) [28] was used to detect viral transcripts in 10X Genomics scRNA-Seq data derived from S1 and S2 PBMC as described in [25]. For this analysis, two distinct consensus sequences of HML-2 (Gene Bank: AB047240) and HML-6 (Gene Bank: AF079797) were used. Integrative Genomics Viewer (IGV) Web App [29] was employed to visualize the respective alignments.

Raw count matrices from S1 and S2 were independently corrected for ambient RNA contamination using SoupX (version 1.6.2) [30] in R, then imported into AnnData objects [31] for downstream analysis using scanpy (version 1.10.2) [32]. Quality control was performed using a multi-layered approach. Minimum thresholds of 200 detected genes per cell and 3 cells per gene were first applied. MAD-based (Median Absolute Deviation) outlier filtering was then performed (5 MADs for gene and UMI counts; 3 MADs for mitochondrial content, with an absolute upper cap of 15%). Cells exceeding 7000 detected genes, 30,000 total UMI counts, 40% ribosomal gene content, or 0.5% hemoglobin gene content were excluded. Putative technical doublets were identified and removed using Scrublet (version 0.2.3) [33], with the expected doublet rate scaled proportionally to the number of cells per sample. Biological doublets were identified by computing lineage-specific gene module scores for T cells, monocytes, NK cells, and B cells using Scanpy’s score_genes function, and removing cells where two incompatible lineage scores simultaneously exceeded a fixed threshold of 0.5. After such filtering steps, 8127 high-quality single-cell transcriptomes were retained. Gene expression counts were normalized to 10,000 counts per cell and log-transformed (log1p). Highly variable genes were identified using Scanpy’s default method. Normalized counts were stored as a dedicated layer and used for all downstream analyses; for dimensionality reduction, they were additionally z-scored (mean = 0, standard deviation = 1, clipped at max_value = 10). Principal Component Analysis (PCA) was performed on the z-scored expression matrix using 50 components, restricted to highly variable genes. Batch correction across samples was performed using Harmony [34] on the PCA embedding. A k-nearest neighbor graph was constructed on the Harmony-corrected embedding and used for both UMAP visualization and Leiden clustering [35], with a resolution of 0.5 that yielded 16 initial clusters. Two clusters containing fewer than 50 cells were removed, resulting in 14 clusters and 8063 cells (S1: 6053 cells; S2: 2010 cells) retained for downstream analysis. One of these clusters that showed transcriptional heterogeneity was further processed by performing subclustering. Cells from this cluster were re-processed independently by performing PCA, k-nearest neighbor graph construction with a number of neighbors of 15 and by considering the first 15 principal components, UMAP embedding, and the Leiden clustering algorithm with a resolution of 0.2.

#### 4.3.2. Cell Type Annotation

Cell type annotation was performed by integrating three complementary approaches: (i) manual annotation based on canonical marker gene expression, validated against published PBMC references [36]; (ii) automated annotation using CellTypist (version 1.6.3) [37], with the Immune_All_High model [38]; (iii) Differential gene expression analysis (DEA) between clusters, performed using the Wilcoxon rank-sum test (one-vs-rest) on log-normalized expression values via scanpy.tl.rank_genes_groups, with genes filtered by minimum log_2_ fold change > 1, minimum detection fraction > 0.25, and maximum out-group detection fraction < 0.5. A monocyte subpopulation with a T cell-like transcriptional signature (IL7R+ Classical Monocytes) was identified within the CD14+ Classical Monocyte cluster based on co-expression of IL7R, CD247, LTB, TCF7, and CCL5, as previously described by Kim et al. 2026 [39]. Although CellTypist classified these cells as CD14^+^ classical monocytes, the low confidence scores (below 0.5) prompted further investigation. The same DEA approach described above, combined with gene module scoring, supported their classification as a distinct subpopulation.

#### 4.3.3. HERV-K Transcript Detection in Single Cells and Enrichment Analysis

Filtered feature-barcode matrices from Cell Ranger runs against HML-2 and HML-6 custom reference genomes were loaded, and cell barcodes were intersected with those retained after quality control, using sample-specific suffixes to ensure unambiguous barcode matching across datasets. For each HERV-K reference and gene segment, UMI counts were mapped to the corresponding cells in the main dataset. Cumulative HERV-K counts were computed by summing UMIs across all gene segments (*gag* and *env* for HML-2; *gag*, *pol*, and *env* for HML-6). A cell was defined as HERV-K-positive if the cumulative UMI count exceeded zero.

### 4.4. HML-6 env Transcripts Quantification in Plasma Samples

Expression levels of HML-6 *env* were established in plasma samples by Real-time PCR, by comparison with the β-actin gene, in 7 acute/recent infections (AHI) and in 7 chronic infections (CHI), at the moment of serodiagnosis (T0) and after 1 year of ART (T1), when HIV-1 RNA levels were below 50 copies/mL in all subjects. Five plasma samples from healthy donors (HD) were used as controls. RNA extraction was performed using QIAmp Viral RNA Kit (QIAGEN, Hilden, Germany). Genomic DNA was removed using RNase-Free DNase Set (QIAGEN) according to the manufacturer’s instructions. cDNA was obtained using SuperScript IV Reverse Transcriptase (ThermoFisher Scientific, Waltham, MA, USA). Real-Time PCR was performed on RotorGene (QIAGEN), using QuantiNova SYBR Green PCR Kit (QIAGEN) to amplify HML-6 *env* and the B-Actin gene in each sample (*env* F: 5′-TGCAAAGACAGAACAAGGGC-3′; *env* R: 5′-CGCACAGGACACTACAGCTA-3′; B-Actin F: 5′-GACCTCTATGCCAACACAGT-3′ and β-Actin R: 5′-AGTACTTGCGCTCAGGAGGA-3′). Amplification conditions were 40 cycles of a two-step cycling protocol, with a denaturation step of 2 min at 95 °C, followed by a combined annealing/extension at 60 °C. To rule out the presence of DNA contamination, amplification of the HML-6 *env* gene was also performed without RT. ΔCt comparative method was used to perform transcript expression analysis of HML-6 env transcripts using β Actin as a reference gene.

### 4.5. Statistics

Enrichment of HERV-K transcripts by cell type was assessed by computing the log_2_ fold change in the mean HERV-K UMI count in each cell type relative to the sample mean. A pseudocount of 1 × 10^−6^ was added to both values prior to log_2_ fold change computation to avoid undefined values. Only cell types with at least one HERV-positive cell were included in this analysis. Statistical significance was determined using a one-sided Mann–Whitney U test, comparing HERV-K UMI counts within each cell type against all remaining cells, with Benjamini–Hochberg FDR correction applied across cell types (adjusted *p* < 0.05). 

Wilcoxon paired test and Mann–Whitney were used to compare differences in HML-6 transcript levels in the two study groups; Spearman correlation test was used to assess correlation between HIV-1 RNA and HML-6 transcript levels; *p* values < 0.05 were considered statistically significant. All statistical tests were carried out using GraphPad Prism v.10.2.1 (GraphPad Software, Boston, MA, USA).

## Figures and Tables

**Table 1:** **Cell type composition of PBMC from S1 and S2.** The first column lists the annotated cell types. The other columns represent the numerosity in S1 and S2, respectively.

Cell Type	S1	S2
T CD8^+^ Tem/Temra	1337	257
T CD8^+^ Teff/NK	841	293
T CD4^+^ naive	567	220
T CD4^+^ Central Memory	546	231
NK cells	212	42
Naive B cells	261	56
Plasma cells	34	68
pDC	42	19
CD14^+^ Classical Monocytes	751	188
IL7R^+^ Classical Monocytes	410	480
CD14^+^ Inflammatory Mono	366	33
CD16^+^ Non-classical Mono	420	34
Low-density granulocytes	218	41
Megakaryocytes/Platelets	48	48
Total	6053	2010

## Data Availability

The raw data supporting the conclusions of this article will be made available by the authors on request.

## Supplementary Materials

The following supporting information can be downloaded at: https://www.mdpi.com/article/10.3390/ijms27136025/s1.

## References

[1] Chen C., Cui Y., Wang S., Yang Y., Liu Z., Jin S., Shen F., Gaipl U., Ma H., Zhou J.-G. (2025). Human Endogenous Retroviruses and Diseases. MedComm.

[2] Viret C., Bynoe M.S. (2024). Human Endogenous Retroviruses Expression in Autoimmunity. Yale J. Biol. Med..

[3] Shah A.H., Govindarajan V., Doucet-O’Hare T.T., Rivas S., Ampie L., DeMarino C., Banasavadi-Siddegowda Y.K., Zhang Y., Johnson K.R., Almsned F. (2022). Differential Expression of an Endogenous Retroviral Element [HERV-K(HML-6)] Is Associated with Reduced Survival in Glioblastoma Patients. Sci. Rep..

[4] Burn A., Roy F., Freeman M., Coffin J.M. (2022). Widespread Expression of the Ancient HERV-K (HML-2) Provirus Group in Normal Human Tissues. PLoS Biol..

[5] Dou A., Xu J., Zhou C. (2025). The Relationship between HERVs and Exogenous Viral Infections: A Focus on the Value of HERVs in Disease Prediction and Treatment. Virulence.

[6] Li M., Yu F., Zhu B., Xiao J., Yan C., Yang X., Liang X., Wang F., Zhang H., Zhang F. (2025). Interactions between Human Immunodeficiency Virus and Human Endogenous Retroviruses. J. Virol..

[7] Gonzalez-Hernandez M.J., Cavalcoli J.D., Sartor M.A., Contreras-Galindo R., Meng F., Dai M., Dube D., Saha A.K., Gitlin S.D., Omenn G.S. (2014). Regulation of the Human Endogenous Retrovirus K (HML-2) Transcriptome by the HIV-1 Tat Protein. J. Virol..

[8] Monde K., Terasawa H., Nakano Y., Soheilian F., Nagashima K., Maeda Y., Ono A. (2017). Molecular Mechanisms by Which HERV-K Gag Interferes with HIV-1 Gag Assembly and Particle Infectivity. Retrovirology.

[9] Gray L.R., Jackson R.E., Jackson P.E.H., Bekiranov S., Rekosh D., Hammarskjöld M.-L. (2019). HIV-1 Rev Interacts with HERV-K RcREs Present in the Human Genome and Promotes Export of Unspliced HERV-K Proviral RNA. Retrovirology.

[10] Stopak K., de Noronha C., Yonemoto W., Greene W.C. (2003). HIV-1 Vif Blocks the Antiviral Activity of APOBEC3G by Impairing Both Its Translation and Intracellular Stability. Mol. Cell.

[11] Neil S.J.D., Zang T., Bieniasz P.D. (2008). Tetherin Inhibits Retrovirus Release and Is Antagonized by HIV-1 Vpu. Nature.

[12] Mantovani F., Kitsou K., Paraskevis D., Lagiou P., Magiorkinis G. (2023). The Interaction of Human Immunodeficiency Virus-1 and Human Endogenous Retroviruses in Patients (Primary Cell Cultures) and Cell Line Models. Microbiol. Spectr..

[13] Grandi N., Pisano M.P., Scognamiglio S., Pessiu E., Tramontano E. (2020). Comprehensive Analysis of HERV Transcriptome in HIV+ Cells: Absence of HML2 Activation and General Downregulation of Individual HERV Loci. Viruses.

[14] Li X., Guo Y., Li H., Huang X., Pei Z., Wang X., Liu Y., Jia L., Li T., Bao Z. (2021). Infection by Diverse HIV-1 Subtypes Leads to Different Elevations in HERV-K Transcriptional Levels in Human T Cell Lines. Front. Microbiol..

[15] Contreras-Galindo R., Kaplan M.H., Contreras-Galindo A.C., Gonzalez-Hernandez M.J., Ferlenghi I., Giusti F., Lorenzo E., Gitlin S.D., Dosik M.H., Yamamura Y. (2012). Characterization of Human Endogenous Retroviral Elements in the Blood of HIV-1-Infected Individuals. J. Virol..

[16] Ormsby C.E., SenGupta D., Tandon R., Deeks S.G., Martin J.N., Jones R.B., Ostrowski M.A., Garrison K.E., Vázquez-Pérez J.A., Reyes-Terán G. (2012). Human Endogenous Retrovirus Expression Is Inversely Associated with Chronic Immune Activation in HIV-1 Infection. PLoS ONE.

[17] Contreras-Galindo R., Almodóvar-Camacho S., González-Ramírez S., Lorenzo E., Yamamura Y. (2007). Short Communication: Comparative Longitudinal Studies of HERV-K and HIV-1 RNA Titers in HIV-1-Infected Patients Receiving Successful versus Unsuccessful Highly Active Antiretroviral Therapy. AIDS Res. Hum. Retrovir..

[18] Chuong E.B., Elde N.C., Feschotte C. (2016). Regulatory Evolution of Innate Immunity through Co-Option of Endogenous Retroviruses. Science.

[19] Ye M., Goudot C., Hoyler T., Lemoine B., Amigorena S., Zueva E. (2020). Specific Subfamilies of Transposable Elements Contribute to Different Domains of T Lymphocyte Enhancers. Proc. Natl. Acad. Sci. USA.

[20] Suligoi B., Regine V., Raimondo M., Rodella A., Terlenghi L., Caruso A., Bagnarelli P., Capobianchi M.R., Zanchetta N., Ghisetti V. (2017). HIV Avidity Index Performance Using a Modified Fourth-Generation Immunoassay to Detect Recent HIV Infections. Clin. Chem. Lab. Med..

[21] Pasternack N., Doucet-O’Hare T., Johnson K., Paulsen O., Nath A. (2024). Endogenous Retroviruses Are Dysregulated in ALS. iScience.

[22] Shah A.H., Rivas S.R., Doucet-O’Hare T.T., Govindarajan V., DeMarino C., Wang T., Ampie L., Zhang Y., Banasavadi-Siddegowda Y.K., Walbridge S. (2023). Human Endogenous Retrovirus K Contributes to a Stem Cell Niche in Glioblastoma. J. Clin. Investig..

[23] Rangel S.C., da Silva M.D., da Silva A.L., Dos de Santos J.M.B., Neves L.M., Pedrosa A., Rodrigues F.M., Trettel C.D.S., Furtado G.E., de Barros M.P. (2022). Human Endogenous Retroviruses and the Inflammatory Response: A Vicious Circle Associated with Health and Illness. Front. Immunol..

[24] Bhardwaj N., Maldarelli F., Mellors J., Coffin J.M. (2014). HIV-1 Infection Leads to Increased Transcription of Human Endogenous Retrovirus HERV-K (HML-2) Proviruses In Vivo but Not to Increased Virion Production. J. Virol..

[25] Pinnetti C., Rozera G., Messina F., Spezia P.G., Lazzari E., Fabeni L., Chillemi G., Pietrucci D., Haggiag S., Mastrorosa I. (2024). Cerebrospinal Fluid and Peripheral Blood Lymphomonocyte Single-Cell Transcriptomics in a Subject with Multiple Sclerosis Acutely Infected with HIV. Int. J. Mol. Sci..

[26] Tyagi R., Li W., Parades D., Bianchet M.A., Nath A. (2017). Inhibition of Human Endogenous Retrovirus-K by Antiretroviral Drugs. Retrovirology.

[27] Ward A.R., Thomas A.S., Stevenson E.M., Huang S.-H., Keating S.M., Gandhi R.T., McMahon D.K., Bosch R.J., Macatangay B.J., Cyktor J.C. (2022). No Evidence That Circulating HIV-Specific Immune Responses Contribute to Persistent Inflammation and Immune Activation in Persons on Long-Term ART. AIDS.

[28] Zheng G.X.Y., Terry J.M., Belgrader P., Ryvkin P., Bent Z.W., Wilson R., Ziraldo S.B., Wheeler T.D., McDermott G.P., Zhu J. (2017). Massively Parallel Digital Transcriptional Profiling of Single Cells. Nat. Commun..

[29] Robinson J.T., Thorvaldsdóttir H., Winckler W., Guttman M., Lander E.S., Getz G., Mesirov J.P. (2011). Integrative Genomics Viewer. Nat. Biotechnol..

[30] Young M.D., Behjati S. (2020). SoupX Removes Ambient RNA Contamination from Droplet-Based Single-Cell RNA Sequencing Data. GigaScience.

[31] Virshup I., Rybakov S., Theis F.J., Angerer P., Wolf F.A. (2024). Anndata: Access and Store Annotated Datamatrices. J. Open Source Softw..

[32] Wolf F.A., Angerer P., Theis F.J. (2018). SCANPY: Large-Scale Single-Cell Gene Expression Data Analysis. Genome Biol..

[33] Wolock S.L., Lopez R., Klein A.M. (2019). Scrublet: Computational Identification of Cell Doublets in Single-Cell Transcriptomic Data. Cell Syst..

[34] Korsunsky I., Millard N., Fan J., Slowikowski K., Zhang F., Wei K., Baglaenko Y., Brenner M., Loh P.-R., Raychaudhuri S. (2019). Fast, Sensitive and Accurate Integration of Single-Cell Data with Harmony. Nat. Methods.

[35] Traag V.A., Waltman L., van Eck N.J. (2019). From Louvain to Leiden: Guaranteeing Well-Connected Communities. Sci. Rep..

[36] Stuart T., Butler A., Hoffman P., Hafemeister C., Papalexi E., Mauck W.M., Hao Y., Stoeckius M., Smibert P., Satija R. (2019). Comprehensive Integration of Single-Cell Data. Cell.

[37] Xu C., Prete M., Webb S., Jardine L., Stewart B.J., Hoo R., He P., Meyer K.B., Teichmann S.A. (2023). Automatic Cell-Type Harmonization and Integration across Human Cell Atlas Datasets. Cell.

[38] Domínguez Conde C., Xu C., Jarvis L.B., Rainbow D.B., Wells S.B., Gomes T., Howlett S.K., Suchanek O., Polanski K., King H.W. (2022). Cross-Tissue Immune Cell Analysis Reveals Tissue-Specific Features in Humans. Science.

[39] Kim D., Choi S.Y., Kim C.Y., Yoo J.R., Kim E.T., Park J. (2026). Discovery of Key Regulators in Classical Monocyte Phenotypes Linked to COVID-19 Severity Using Single-Cell Multi-Omics Sequencing. iScience.

