# Supplementary material for: Expression of Human Endogenous Retroviruses in Peripheral Blood of Acute and Chronically HIV-Infected Subjects and Effect of Antiretroviral Therapy
Source: Int J Mol Sci. 2026 Jul 4;27(13):6025. doi: 10.3390/ijms27136025 (PMC13361886; doi:10.3390/ijms27136025)
Supplement: Supplementary file 1 [file ijms-27-06025-s001.zip › ijms-4376732-supplementary.pdf]

	Total number of Reads	Mean Reads per Cell	Number of viral-mapped reads	Number of mapped reads with MAPQ =255
S1_HML_2	4,696,375	52,768	13,986	4,172
S2_HML_2	16,745,367	322,026	10,143	4,020
S1_HML_6	4,696,375	8,844	31,476	15,346
S2_HML_6	16,745,367	47,844	35,010	19,085

Supplementary Table S1. Sequencing depth and alignment metrics for HERV-K reads across S1 and S2 subjects. For each sample (S1 and S2) aligned to HERV-K HML-2 (Gene Bank: AB047240) and HML-6 (Gene Bank: AF079797) genomes the table reports: the total number of unmapped human reads used as input for the viral alignments, the mean reads per cell, the total number of reads mapped to the viral reference genome, and the total number of uniquely aligned reads (MAPQ =255).
